# Endogenous biotin-binding proteins: an overlooked factor causing false positives in streptavidin-based protein detection

**DOI:** 10.1111/1751-7915.12150

**Published:** 2014-09-11

**Authors:** Hanne L P Tytgat, Geert Schoofs, Michèle Driesen, Paul Proost, Els J M Van Damme, Jos Vanderleyden, Sarah Lebeer

**Affiliations:** 1 Department of Bioscience Engineering, Research Group Environmental Ecology and Applied Microbiology, University of AntwerpAntwerp, Belgium; 2 Department of Microbial and Molecular Systems, Centre of Microbial and Plant GeneticsLeuven, Belgium; 3 Department of Microbiology and Immunology, Laboratory of Molecular Immunology, KU LeuvenLeuven, Belgium; 4 Department of Molecular Biotechnology, Laboratory of Biochemistry and Glycobiology, Ghent UniversityGhent, Belgium

## Abstract

Biotinylation is widely used in DNA, RNA and protein probing assays as this molecule has generally no impact on the biological activity of its substrate. During the streptavidin-based detection of glycoproteins in *Lactobacillus rhamnosus* GG with biotinylated lectin probes, a strong positive band of approximately 125 kDa was observed, present in different cellular fractions. This potential glycoprotein reacted heavily with concanavalin A (ConA), a lectin that specifically binds glucose and mannose residues. Surprisingly, this protein of 125 kDa could not be purified using a ConA affinity column. Edman degradation of the protein, isolated via cation and anion exchange chromatography, lead to the identification of the band as pyruvate carboxylase, an enzyme of 125 kDa that binds biotin as a cofactor. Detection using only the streptavidin conjugate resulted in more false positive signals of proteins, also in extracellular fractions, indicating biotin-associated proteins. Indeed, biotin is a known cofactor of numerous carboxylases. The potential occurence of false positive bands with biotinylated protein probes should thus be considered when using streptavidin-based detection, e.g. by developing a blot using only the streptavidin conjugate. To circumvent these false positives, alternative approaches like detection based on digoxigenin labelling can also be used.

## Introduction

Biotin is a small molecule that is widely used in molecular biology as a result of its extremely high affinity for streptavidin binding (K_d_ = 10^−14^–10^−15^ M) (Green, 1975; Laitinen *et al*., 2006). The streptavidin–biotin interaction is one of the strongest non-covalent bonds known in nature, in strength almost matching covalent strength (Chaiet and Wolf, 1964; Laitinen *et al*., 2006). The rigid nature of the bond results in resistance of the complex against organic solvents, denaturants, detergents, proteolytic enzymes and extreme pH and temperature conditions. This is exploited in biological assays by coupling biotin to substrates like DNA, RNA and proteins. Detection and purification of these substrates is then accomplished by application of a streptavidin conjugate tagged with an enzyme reporter (like alkaline phosphatase or horseradish peroxidase) or a fluorescent probe (Chevalier *et al*., 1997; Chapman-Smith and Cronan, 1999). Another important advantage of this detection method is the low molecular weight of the biotin, which enables linkage to its substrate without affecting the substrate activity. To further limit effects on substrate activity, the biotin is mostly linked via an extending long linker (Chevalier *et al*., 1997; Chapman-Smith and Cronan, 1999).

Biotin is an important cofactor of carboxylase enzymes in all domains of life. The biotin cofactor is involved in the transfer of carbon dioxide in oxidative metabolism reactions. Biotin is bound via a biotin carboxyl carrier protein that can be part of a multidomain carboxylase or substitute a separate subunit of the carboxylase enzyme complex (Wood and Barden, 1977; Fugate and Jarrett, 2012; Tong, 2013). Some of these proteins can be moonlighting proteins, as described in yeast (Huberts *et al*., 2010).

Earlier reports already mentioned the presence of proteins binding biotin as a cofactor resulting from false positive reactions during gene probing in bacteria (Wang *et al*., 1993), and several reports have been published on the interference of endogenous biotin in eukaryotic assays (McKay *et al*., 2008; Chen *et al*., 2012; Horling *et al*., 2012). Nevertheless, many research groups still use biotinylated probes to detect specific DNA, RNA and protein structures. With the booming interest in glycobiology and bacterial glycoproteins, biotinylated lectins are now widely used to identify specific sugar residues (Coyne *et al*., 2005; Lebeer *et al*., 2012; Lee *et al*., 2014; Wu *et al*., 2014). We report here on the occurrence of false positive hits due to endogenous biotin-binding proteins when biotinylated probes are used to probe (glyco-)proteins.

In this study, we aimed at the detection of specific sugar modifications on proteins of *Lactobacillus rhamnosus* GG using biotinylated lectins. Intriguingly, an apparent strong positive protein band of approximately 125 kDa present in several proteome fractions could not be purified using lectin affinity chromatography. The protein turned out to be LGG_01329, a pyruvate carboxylase that harbours a domain that binds a biotin cofactor. This protein did not stain with the glycoprotein Periodic Acid Schiff (PAS) base staining method, and is thus not a glycoprotein. Probing the protein samples using only streptavidin lead to the discovery of even more false positive hits. We therefore suggest the implementation of alternatives such as the digoxigenin detection method to circumvent these false hits.

## Results and discussion

### Detection of an intriguing 125 kDa band using biotinylated lectins

The discovery of a glycosylated protein in the model probiotic *L. rhamnosus* GG (ATCC 53103) (Lebeer *et al*., 2012) raised the question if this bacterium is able to produce more glycoproteins. Therefore, a Western blot screening was designed in which an array of biotinylated lectins was used to sample the glycosylation state of the proteome of *L. rhamnosus* GG. Lectins are (glyco-)proteins which, similar to the use of antibodies in Western blot development, can bind specific carbohydrate structures. Their binding affinity depends on the configuration and accessibility of the specific sugar(s) that are recognized by the lectins under study (Van Damme *et al*., 2011).

**Fig 1:**
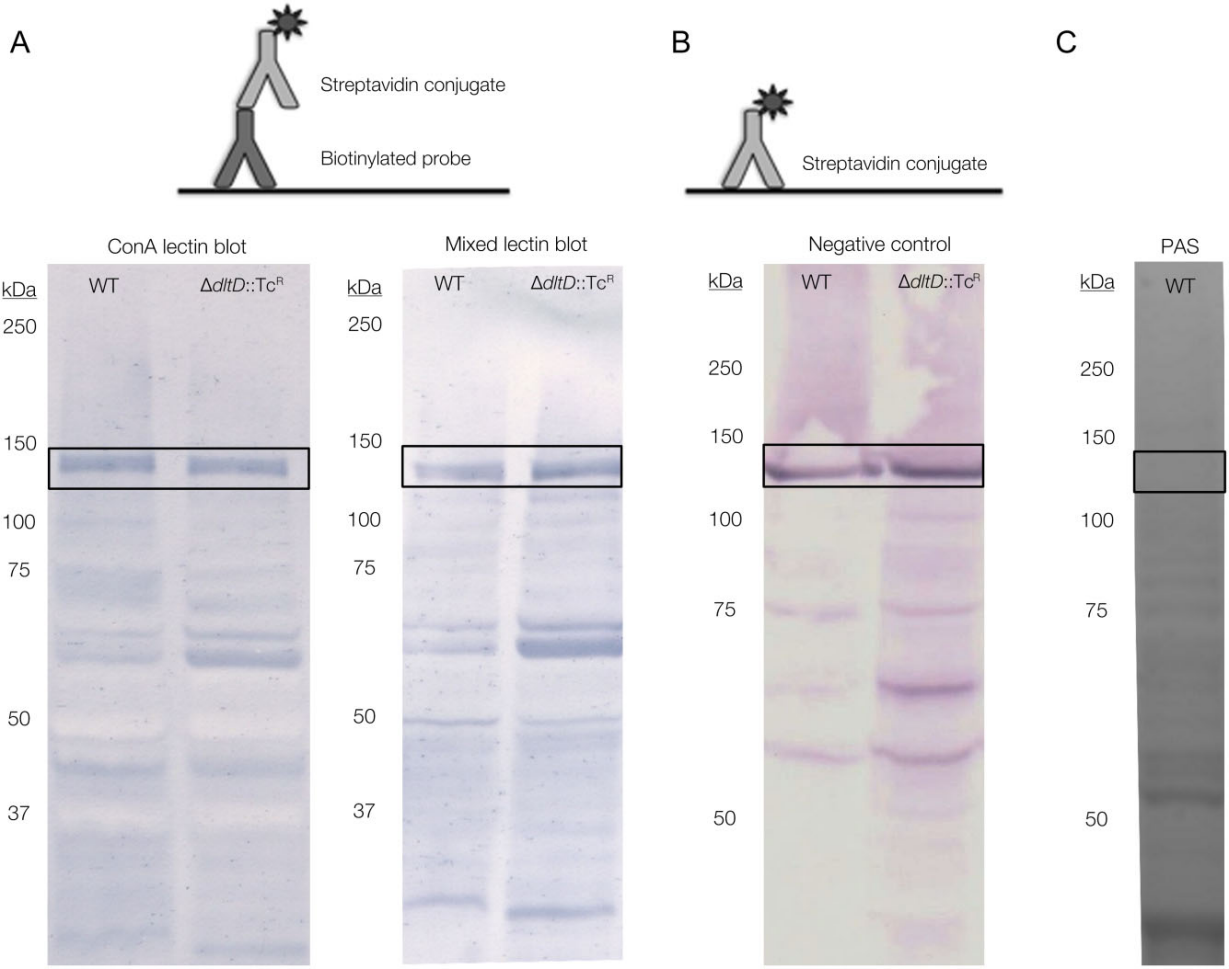
Detection of glycoproteins on Western blot using biotinylated lectins and streptavidin results in false positive hits.A. Biotinylated lectin blots – The exoproteome of wild type *L. rhamnosus* GG and its Δ*dltD*::Tc^R^ mutant were subjected to SDS-PAGE on NuPAGE® Novex® 12% Bis-Tris gels (Life Technologies) and subsequently blotted to PVDF membranes (Life Technologies). The prestained Kaleidoscope™ ladder (Bio-Rad) was added as a molecular weight marker. The Western blots were developed using biotinylated probes, in this case lectins. In the left panel, the Western blot was probed with biotinylated ConA, which specifically binds glucose and terminal mannose. Incubation with streptavidin conjugated to alkaline phosphatase (Roche) enabled the visual detection of positive bands using NBT and BCIP. In the right panel, the blot was developed using the same principle, but initial probing was performed using a mix of biotinylated lectins: ConA (Glc, Man), GNA (Man), HHA (Man), WGA (GlcNAc), DSL (GlcNAc), UDA (GlcNAc), Nictaba (GlcNAc), RSA (Gal, GalNAc) and PNA (Gal, GalNAc). In both Western blots and for both strains, a peculiarly strong band appeared at approximately 125 kDa.B. Using a streptavidin conjugate to sample the proteome for false positive hits – The blotted exoproteome of *L. rhamnosus* GG and the Δ*dltD*::Tc^R^ mutant were probed directly with a streptavidin conjugate. This resulted in the appearance of several bands, among which the strong 125 kDa band. Based on this result, we suggest that the band is not caused by a glycoprotein, but is a false positive.C. PAS glycostain does not react with the 125 kDa protein – An SDS-PAGE gel of the proteome of *L. rhamnosus* GG was post-stained with Periodic Acid Schiff base stain (PAS, Pro-Q® Emerald 488 stain, Life Technologies), a method to specifically stain glycosylated proteins in a gel. At 125 kDa, no band could be detected, which further supports our hypothesis that the 125 kDa signal perceived on the lectin blots is the result of a false positive hit.

We applied an array of biotinylated lectins with different specificities to Western blots of proteome samples of *L. rhamnosus* GG to screen for glycosylated proteins. We here depict the results (Fig. 1A) for Western blots of the extracellular proteome developed using concanavalin A (ConA) (specific for glucose, terminal mannose) and a mix of lectins consisting out of ConA, the *Galanthus nivalis* agglutinin (GNA; terminal mannose), the *Hippeastrum* hybrid agglutinin (HHA; mannose), Wheat germ agglutinin (WGA) [*N-*acetylglucosamine (GlcNAc)], DSL, a lectin isolated from *Datura stramonium* (GlcNAc), UDA, an agglutinin originating from *Urtica dioica* (GlcNAc), Nictaba purified from *Nicotiana tabacum* leaves (GlcNAc), the *Rhizoctonia solani* agglutinin (RSA) [Galactose (Gal), *N-*acetylgalactosamine (GalNAc)] and PNA or peanut agglutinin (Gal, GalNAc). Figure 1A shows the resulting lectin blots for the proteome of wild type *L. rhamnosus* GG and the Δ*dltD*::Tc^R^ mutant (CMPG5540) (Perea Velez *et al*., 2007). After incubation with the biotinylated lectins, the Western blots were probed with a streptavidin conjugate that enabled the visual detection of bands (Fig. 1A).

To our surprise, both wild type and the Δ*dltD*::Tc^R^ mutant samples showed a strong band at approximately 125 kDa (Fig. 1A). All repetitions of the assays using single biotinylated lectins or mixtures of lectins confirmed these findings (results not shown). The same 125 kDa band was also observed on Western blots of the proteomes of other mutants of *L. rhamnosus* GG as well as in the probing of other cellular proteomic fractions (results not shown).

### Purification of the 125 kDa protein

We set out to identify the protein eliciting this strong signal on all blots. Based on the positive signal of the protein with ConA, a ConA affinity chromatography approach (HiTrap ConA 4B column, GE Healthcare) was initiated. However, despite of numerous efforts, we were unable to purify the protein in this set-up. Finally, a combination of cation (SP Sepharose HP, GE Healthcare) and anion exchange chromatography (Q Sepharose HP, GE Healthcare) resulted in the successful purification of the protein. Identification by Edman degradation on a Procise 491 cLC protein sequencer revealed that the LGG_01329 protein, a pyruvate carboxylase, is responsible for the positive bands on the lectin blots. The theoretical molecular weight of 125028 Da for this protein corresponds to the apparent molecular weight observed on SDS-PAGE (results not shown) and on the lectin blots (Fig. 1A). The molecular weight of glycosylated proteins typically differs from the mass calculated from the deduced amino acid sequence of the protein, which forms a first indication that the protein encoded by *LGG_01329* is not glycosylated. This was confirmed with a gel stained with PAS base stain (Pro-Q® Emerald 488 stain, Life Technologies), a glycoprotein stain, on which the 125 kDa band is absent (Fig. 1C). Taken together with the fact that carboxylases bind endogenous biotin as a cofactor, we suggest that the LGG_01329 protein is a false positive hit resulting from the reaction of the endogenous biotin with the streptavidin detection method, even when the extracellular proteome is investigated. This also would explain why the 125 kDa protein could not be purified using ConA affinity chromatography, despite of its strong positive reaction with biotinylated ConA on Western blot.

### Confirmation that false positives are caused by endogenous biotin

To further confirm our hypothesis that the false positive 125 kDa band resulted from the binding of endogenous biotin to the pyruvate carboxylase encoded by *LGG_01329*, we performed a Western blot on the extracellular proteome fraction in which we probed the blot with streptavidin alone. This means that we did not apply any primary biotinylated protein (in this case lectins), but merely developed the blot after incubation with streptavidin conjugate (Fig. 1B). These results are depicted in Fig. 1B, in which a Western blot of the exoproteome of wild type *L. rhamnosus* GG and the Δ*dltD*::Tc^R^ mutant is shown, which was probed with streptavidin. One of the strongest signals on this blot could be found at 125 kDa (Fig. 1B) and represents the pyruvate carboxylase LGG_01329.

The streptavidin blot also reveals several other bands, which represent other false positives. Indeed, several genes turn up when screening the genome of *L. rhamnosus* GG for proteins binding biotin or related to biotin metabolism (Gene database of NCBI). Examples are the AccC (LGG_02112) and AccB (LGG_02114) proteins encoding an Acetyl-CoA carboxylase biotin carboxylase and its biotin carboxyl carrier protein subunit respectively.

These findings implicate that the interpretation of Western blots developed with biotinylated probes should be carried out with caution. We therefore suggest to always include a control Western blot only probed with streptavidin conjugate when using biotinylated probes (Fig. 1B). Screening of the genome of the species under study can also reveal the presence of biotin-binding enzymes.

**Fig 2:**
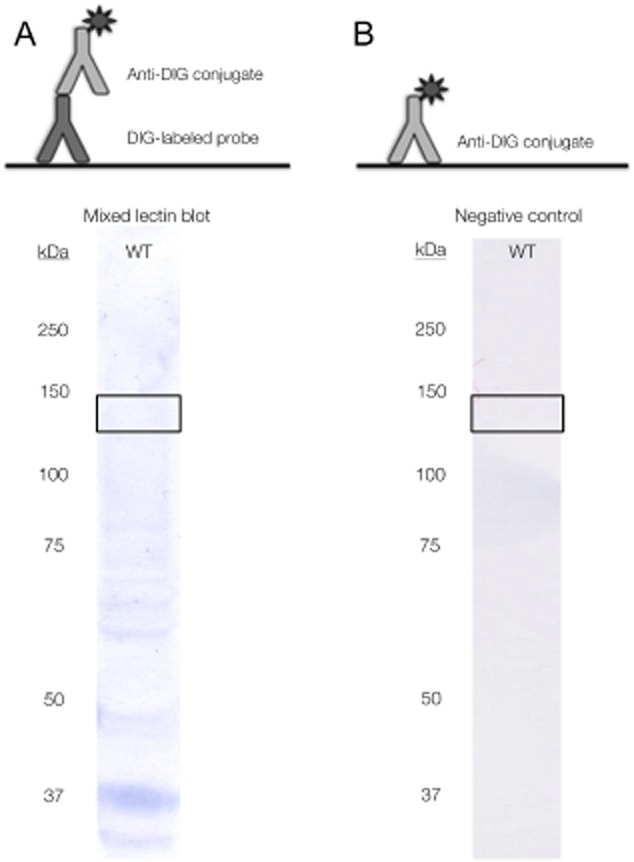
The digoxigenin–anti-digoxigenin detection as an alternative to avoid false positive hits caused by proteins binding endogenous biotin.A. DIG-labelled lectin blots – The wild type exoproteome of *L. rhamnosus* GG was Western blotted and developed using a mix of DIG-labelled lectins: ConA (Glc, Man), GNA (Man), HHA (Man), WGA (GlcNAc), DSL (GlcNAc), UDA (GlcNAc), Nictaba (GlcNAc), RSA (Gal, GalNAc) and PNA (Gal, GalNAc). These lectins were labelled using digoxigenin-3-O-methyl-ε-aminocaproix acid-N-hydroxysuccinimide ester (Roche). Anti-DIG Fab antibody fragments (Roche) were used to detect proteins that reacted positively with the lectin probes. Here, we clearly see that the false positive band at 125 kDa is absent.B. Negative control with anti-DIG – Direct application of the anti-DIG Fab antibody fragments (Roche) to the Western blotted proteome of *L. rhamnosus* GG results in a blot on which no bands can be perceived. This confirms that the DIG–anti-DIG detection method is a good alternative for the biotin–streptavidin system, without causing false positive hits.

To circumvent the occurrence of false positive bands completely, we suggest the digoxigenin–anti-digoxigenin (DIG–anti-DIG) detection method as a good alternative. As digoxigenin is a steroid only produced by *Digitalis* plants, interference of endogenous material in other species is not an issue (Chevalier *et al*., 1997) (cf. Fig. 2B, for a confirmation in *L. rhamnosus* GG). The DIG label is recognized by an anti-DIG antibody that binds with a high specificity to the small steroid molecule (only 390 Da). In practical set-ups, only the Fab fragments of the anti-DIG antibody are used to avoid (albeit rare) cross reaction with structurally related steroids (Kessler, 1991). An N-hydroxysuccinimide ester derivative with a 6-aminocaproate spacer is commercially available to label probes with DIG. Results for the *L. rhamnosus* GG wild type exoproteome probed with a DIG-labelled lectin mix show less bands and most importantly, at 125 kDa, no band is visible, which leads to the presumption that false positives hits are lacking (Fig. 2A). This hypothesis is confirmed by a Western blot developed using only the anti-DIG antibody (Fig. 2B).

Our findings are corroborated by earlier reports on false positive results caused by endogenous biotin in eukaryotes (McKay *et al*., 2008; Chen *et al*., 2012; Horling *et al*., 2012) and in the gene probing of *Streptococcus mutans* (Wang *et al*., 1993). Interestingly, recent work by Lee *et al*. on the glycoproteome of *L. plantarum* WCFS-1 reported a recurring band of approximately 125 kDa on Western blots developed with the biotinylated WGA and the biotinylated lectins of *Dolichos biflorus* and *Lens culinaris* (Lee *et al*., 2014). Based on our results, we suggest that this band could correspond to the pyruvate carboxylase Lp_2136 of 127 kDa.

## Conclusion

In this work, we illustrated the occurrence of false positive results on Western blots developed with biotinylated probes. Biotin-binding proteins, such as carboxylases, which bind endogenous biotin as a cofactor, cause these false positives. These proteins also occur in extracellular fractions, possibly as moonlighting proteins. Based on the here-presented results, we suggest that when using biotinylated probes, a control experiment only using a streptavidin probe should be included (Fig. 1B). A good alternative labelling method is DIG labelling and detection with anti-DIG Fab fragments.

## Conflict of interest

None declared.

## References

[b1] Chaiet L, Wolf FJ (1964). The properties of streptavidin, a biotin-binding protein produced by streptomycetes. Arch Biochem Biophys.

[b2] Chapman-Smith A, Cronan JE (1999). Molecular biology of biotin attachment to proteins. J Nutr.

[b3] Chen T, Hedman L, Mattila PS, Jartti L, Jartti T, Ruuskanen O (2012). Biotin IgM antibodies in human blood: a previously unknown factor eliciting false results in biotinylation-based immunoassays. PLoS ONE.

[b4] Chevalier J, Yi J, Michel O, Tang XM (1997). Biotin and digoxigenin as labels for light and electron microscopy in situ hybridization probes: where do we stand?. J Histochem Cytochem.

[b5] Coyne MJ, Reinap B, Lee MM, Comstock LE (2005). Human symbionts use a host-like pathway for surface fucosylation. Science.

[b6] Fugate CJ, Jarrett JT (2012). Biotin synthase: insights into radical-mediated carbon-sulfur bond formation. Biochim Biophys Acta.

[b7] Green NM (1975). Avidin. Adv Protein Chem.

[b8] Horling L, Neuhuber WL, Raab M (2012). Pitfalls using tyramide signal amplification (TSA) in the mouse gastrointestinal tract: endogenous streptavidin-binding sites lead to false positive staining. J Neurosci Methods.

[b9] Huberts DH, Venselaar H, Vriend G, Veenhuis M, van der Klei IJ (2010). The moonlighting function of pyruvate carboxylase resides in the non-catalytic end of the TIM barrel. Biochim Biophys Acta.

[b10] Kessler C (1991). The digoxigenin:anti-digoxigenin (DIG) technology – a survey on the concept and realization of a novel bioanalytical indicator system. Mol Cell Probes.

[b11] Laitinen OH, Hytonen VP, Nordlund HR, Kulomaa MS (2006). Genetically engineered avidins and streptavidins. Cell Mol Life Sci.

[b12] Lebeer S, Claes IJ, Balog CI, Schoofs G, Verhoeven TL, Nys K (2012). The major secreted protein Msp1/p75 is O-glycosylated in *Lactobacillus rhamnosus* GG. Microb Cell Fact.

[b13] Lee IC, van Swam II, Tomita S, Morsomme P, Rolain T, Hols P (2014). GtfA and GtfB are both required for protein O-glycosylation in Lactobacillus plantarum. J Bacteriol.

[b14] McKay BE, Molineux ML, Turner RW (2008). Endogenous biotin in rat brain: implications for false-positive results with avidin-biotin and streptavidin-biotin techniques. Methods Mol Biol.

[b15] Perea Velez M, Verhoeven TL, Draing C, Von Aulock S, Pfitzenmaier M, Geyer A (2007). Functional analysis of D-alanylation of lipoteichoic acid in the probiotic strain *Lactobacillus rhamnosus* GG. Appl Environ Microbiol.

[b16] Tong L (2013). Structure and function of biotin-dependent carboxylases. Cell Mol Life Sci.

[b17] Van Damme EJ, Smith DF, Cummings R, Peumans WJ (2011). Glycan arrays to decipher the specificity of plant lectins. Adv Exp Med Biol.

[b18] Wang D, Waye MM, Taricani M, Buckingham K, Sandham HJ (1993). Biotin-containing protein as a cause of false positive clones in gene probing with streptavidin/biotin. Biotechniques.

[b19] Wood HG, Barden RE (1977). Biotin enzymes. Annu Rev Biochem.

[b20] Wu J, Zhu J, Yin H, Buckanovich RJ, Lubman DM (2014). Analysis of glycan variation on glycoproteins from serum by the reverse lectin-based ELISA assay. J Proteome Res.

